# Generalised Measures of Multivariate Information Content

**DOI:** 10.3390/e22020216

**Published:** 2020-02-14

**Authors:** Conor Finn, Joseph T. Lizier

**Affiliations:** 1 Centre for Complex Systems, The University of Sydney, Sydney NSW 2006, Australia; joseph.lizier@sydney.edu.au; 2 CSIRO Data61, Marsfield NSW 2122, Australia

**Keywords:** information content, multivariate mutual information, information measures, information decomposition, synergy, redundancy

## Abstract

The entropy of a pair of random variables is commonly depicted using a Venn diagram. This representation is potentially misleading, however, since the multivariate mutual information can be negative. This paper presents new measures of multivariate information content that can be accurately depicted using Venn diagrams for any number of random variables. These measures complement the existing measures of multivariate mutual information and are constructed by considering the algebraic structure of information sharing. It is shown that the distinct ways in which a set of marginal observers can share their information with a non-observing third party corresponds to the elements of a free distributive lattice. The redundancy lattice from partial information decomposition is then subsequently and independently derived by combining the algebraic structures of joint and shared information content.

## 1. Introduction

For any pair of random variables *X* and *Y*, the entropy *H* satisfies the inequality

(1) H(X)+H(Y)≥H(X,Y)≥H(X),H(Y)≥0.

From this inequality, it is easy to see that the conditional entropies and mutual information are non-negative,

(2) $$\begin{array}{cc} H(X|Y) & =H(X,Y)-H(Y)\geq 0, \end{array}$$

(3) $$\begin{array}{cc} H(Y|X) & =H(X,Y)-H(X)\geq 0, \end{array}$$

(4) $$\begin{array}{cc} I(X;Y) & =H(X)+H(Y)-H(X,Y)\geq 0. \end{array}$$

For any pair of sets *A* and *B*, a measure μ satisfies the inequality

(5) μ(A)+μ(B)≥μ(A∪B)≥μ(A),μ(B)≥0,

which follows from the non-negativity of measure on the relative complements and the intersection,

(6) $$\begin{array}{cc} \mu (A\backslash B) & =\mu (A\cup B)-\mu (B)\geq 0 \end{array}$$

(7) $$\begin{array}{cc} \mu (B\backslash A) & =\mu (A\cup B)-\mu (A)\geq 0 \end{array}$$

(8) $$\begin{array}{cc} \mu (A\cap B) & =\mu (A)+\mu (B)-\mu (A\cup B)\geq 0. \end{array}$$

**Figure 1:**
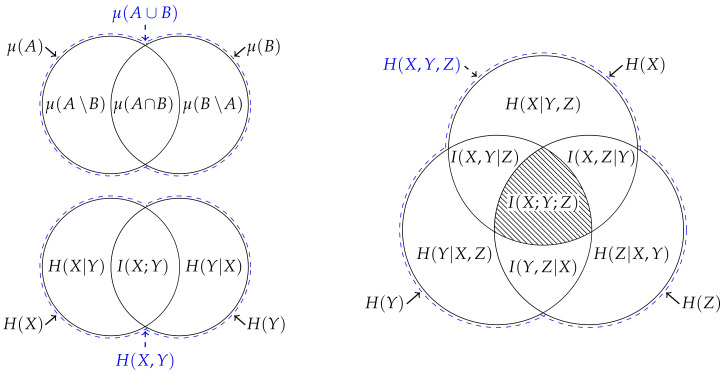
(**Top left**) When depicting a measure on the union of two sets μ(A∪B), the area of each section can be used to represent the inequality (5) and hence the values μ(A\B), μ(B\A) and μ(A∩B) correspond to the area of each section. This correspondence can be generalised to consider an arbitrary number of sets. (**Bottom left**) When depicting the joint entropy H(X,Y), the area of each section can also be used to represent the inequality (1) and hence the values H(X|Y), H(Y|X) and I(X;Y) correspond to the area of each section. However, this correspondence does not generalise beyond two variables. (**Right**) For example, when considering the entropy of three variables, the multivariate mutual information I(X;Y;Z) cannot be accurately represented using an area since, as represented by the hatching, it is not non-negative.

Although the entropy is not itself a measure, several authors have noted the entropy is analogous to measure in this regard [1,2,3,4,5,6,7]. Indeed, it is this analogy which provides the justification for the typical depiction of a pair of entropies using Venn diagrams, i.e., Figure 1. Nevertheless, MacKay [8] noted that this representation is misleading for at least two reasons: Firstly, since the measure on the intersection μ(A∩B) is a measure on a set, it gives the false impression that the mutual information I(X;Y) is the entropy of some intersection between the random variables. Secondly, it might lead one to believe that this analogy can be generalised beyond two variables. However, the analogy does not generalise beyond two variables since the multivariate mutual information [9] between three random variables (which is also known as the interaction information [10], amount of information [2] or co-information [11]),

(9) $$\begin{array}{cc} I(X;Y;Z) & =H(X)+H(Y)+H(Z)-H(X,Y)-H(X,Z)-H(Y,Z)+H(Z,Y,Z), \end{array}$$

is not non-negative [3,9,12], and hence is not analogous to measure on the triple intersection μ(A∩B∩C) [3]. Indeed, this “unfortunate” property led Cover and Thomas to conclude that “there isn’t really a notion of mutual information common to three random variables” (p. 49 [13]). Consequently, MacKay [8] recommended against depicting the entropy of three or more variables using a Venn diagram, i.e., Figure 1, unless one is aware of these issues with this representation.

However, Yeung [6,7] showed that there is an analogy between entropy and *signed* measure that is valid for an arbitrary number of random variables. To do this, Yeung defined a signed measure on a suitably constructed σ-algebra that is uniquely determined by the joint entropies of the random variables involved. This correspondence enables one to establish information-theoretic identities from measure-theoretic identities and hence Venn diagrams can be used to represent the entropy of three or more variables provided one is aware that the certain overlapping areas may correspond to negative quantities. Moreover, the multivariate mutual information is useful both as summary quantity and for manipulating information-theoretic identities provided one is mindful it may have “no intuitive meaning” [5,6].

In this paper, we introduce new measures of multivariate information that are analogous to measures upon sets and maintain their operational meaning when considering an arbitrary number of variables. These new measures complement the existing measures of multivariate mutual information, and will be constructed by considering the distinct ways in which a set of marginal observers might share their information with a non-observing third party. In Section 2, we discuss the existing measures of information content in terms of a set of individuals who each have different knowledge about a joint realisation from a pair of random variables. Then, in Section 3, we discuss how these individuals can share their information with a non-observing third party, and derive the functional form of this individual’s information. In Section 4, we relate this new measure of information content back to the mutual information. Section 5, Section 6 and Section 7 then generalise the arguments of Section 3 and Section 4 to consider an arbitrary number of observers. Finally, in Section 8, we discuss how these new measures can be combined to define new measures of mutual information.

## 2. Mutual Information Content

Suppose that Alice and Bob are separately observing some process and let the discrete random variables *X* and *Y* represent their respective observations. Say that Johnny is a third individual who can simultaneously make the same observations as Alice and Bob such that his observations are given by the joint variable (X,Y). When a realisation (x,y) occurs, Alice’s information is given by the information content [8],

(10) $$h\left(x\right)=-\log p_{X}\left(x\right)\geq 0,$$

where $p_{X}\left(x\right)$ is the probability mass of the realisation *x* of variable *X* computed from the probability distribution $p_{X}$. Likewise, Bob’s information is given by the information content h(y), while Johnny’s information is by the joint information content $h\left(x,y\right)=-\log p_{XY}\left(x,y\right)$. The information that Alice can expect to gain from an observation is given by the entropy,

(11) $$H\left(X\right)=E_{X}\left[h(x)\right]\geq 0,$$

where $E_{X}$ represents an expectation value over realisations of the variable *X*. Similarly, Bob’s expected information gain is given by the entropy H(X) and Johnny’s expected information is given by the joint entropy $H\left(X,Y\right)=E_{XY}\left[h\left(x,y\right)\right]$. Clearly, for any realisation, Johnny has at least as much information as either Alice or Bob,

(12) h(x,y)≥h(x),h(y)≥0.

The conditional information content can be used to quantify how much more information Johnny has relative to either Alice or Bob, respectively,

(13) $$\begin{array}{ccc} h(x|y) & =h(x,y)-h(y) & \geq 0, \end{array}$$

(14) $$\begin{array}{ccc} h(y|x) & =h(x,y)-h(x) & \geq 0. \end{array}$$

Similarly, we can quantify how much more information Johnny expects to get compared to either Alice or Bob via the conditional entropies,

(15) $$\begin{array}{ccc} H(X|Y) & =E_{XY}\left[h(x|y)\right] & \geq 0, \end{array}$$

(16) $$\begin{array}{ccc} H(Y|X) & =E_{XY}\left[h(y|x)\right] & \geq 0. \end{array}$$

Now, consider a fourth individual who does not directly observe the process, but with whom Alice and Bob share their knowledge. To be explicit, we are considering the situation whereby this individual knows that the joint realisation (x,y) has occurred and knows the marginal distributions $p_{X}$ and $p_{Y}$, but does not know the joint distribution $p_{XY}$. How much information does this individual obtain from the shared marginal knowledge provided by Alice and Bob? The answer to this question is provided in Section 3, but for now let us consider a simplified version of this problem. Suppose that such an individual, whom we call Indiana (or Indy for short), assumes that Alice’s observations are independent of Bob’s observations. In terms of the probabilities, this means that Indy believes that the joint probability $p_{XY}\left(x,y\right)$ is equal to the product probability $p_{X\times Y}\left(x,y\right)=p_{X}\left(x\right)\;p_{Y}\left(y\right)$, while, in terms of information, this assumption leads Indiana to believe that her information is given by the independent information content h(x)+h(y). Moreover, the information that Indiana expects to gain from any one realisation is given by H(X)+H(Y).

Let us now compare how much information Indiana believes that she has compared to our other observers. For every realisation, Indiana believes that she has at least as much information as either Alice or Bob,

(17) h(x)+h(y)≥h(x),h(y)≥0.

**Figure 2:**
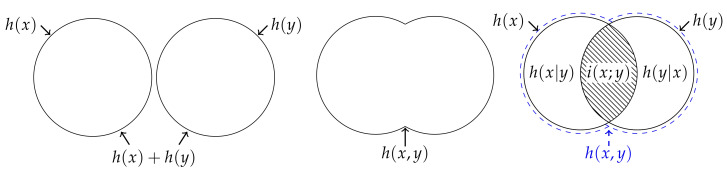
(**Left**) Indiana assumes that Alice’s information h(x) is independent of Bob’s information h(y) such that her information is given by h(x)+h(y). (**Middle**) Johnny knows the joint distribution $p_{XY}$, and hence his information is given by the joint information content h(x,y). (**Right**) There is no inequality that requires Johnny’s information to be no greater than Indiana’s assumed information, or vice versa. On the one hand, Johnny can have more information than Indiana since a joint realisation can be more surprising than both of the individual marginal realisations. On the other hand, Indiana can have more information than Johnny since a joint realisation can be less surprising than both of the individual marginal realisations occurring independently. Thus, as represented by the hatching, the mutual information content i(x;y) is not non-negative.

Since Indy knows what both Alice and Bob know individually, it is hardly surprising that she always has at least as much information as either Alice or Bob. The comparison between Indiana and Johnny, however, is not so straightforward—there is no inequality that requires the information content of the joint realisation to be less than the information content of the independent realisations, or vice versa. Consequently, the difference between the information that Indiana thinks she has and Johnny’s information, i.e., the mutual information content between a pair of realisations,

(18) $$i\left(x;y\right)=h\left(x\right)+h\left(y\right)-h\left(x,y\right)=\log\frac{p_{XY}\left(x,y\right)}{p_{X}\left(x\right)\;p_{Y}\left(y\right)},$$

is not non-negative [14]. (This function goes by several different names including the pointwise mutual information, the information density [15] or simply the mutual information [9].) Thus, similar to how it is potentially misleading to depict the entropy of three of more variables using a Venn diagram, representing the information content of two variables using a Venn diagram is somewhat dubious (see Figure 2).

Since Johnny knows the joint distribution $p_{XY}$, while Indiana only knows the marginal distributions $p_{X}\left(x\right)$ and $p_{Y}\left(y\right)$, we might expect that Indiana should never have more information than Johnny. However, Indiana’s assumed information is based upon the belief that Alice’s observations *X* are independent of Bob’s observations *Y*, which leads Indiana to overestimate her information on average. Indeed, Indiana is so optimistic that the information she expects to get upper bounds the information that Johnny can expect to get,

(19) H(X)+H(Y)≥H(X,Y)≥0.

Thus, despite the fact that Indiana can have less information than Johnny for certain realisations—i.e., despite the fact that the mutual information content is not non-negative—the mutual information in expectation is non-negative,

(20) $$I\left(X;Y\right)=H\left(X\right)+H\left(Y\right)-H\left(X,Y\right)=E_{XY}\left[i(x;y)\right]\geq 0.$$

Crucially, and in contrast to the information content (10) and entropy (11), the non-negativity of the mutual information does not follow directly from the non-negativity of the mutual information content (18), but rather must be proved separately. (Typically, this is done by showing that the mutual information can be written as a Kullback–Leibler divergence which is non-negative by Jensen’s inequality, e.g., see Cover and Thomas [13].) Thus, not only does Indiana potentially have more information than Johnny for certain realisations, but on average we expect Indiana to have more information than Johnny. Of course, by assuming Alice’s observations are independent of Bob’s observations, Indiana is overestimating her information. Thus, in the next section, we consider the situation whereby one does not make this assumption.

## 3. Marginal Information Sharing

Suppose that Eve is another individual who, similar to Indiana, does not make any direct observations, but with whom both Alice and Bob share their knowledge; i.e., Eve knows the joint realisation (x,y) has occurred and knows the marginal distributions $p_{X}$ and $p_{Y}$, but does not know the joint distribution $p_{XY}$. Furthermore, suppose that Eve is more conservative than Indiana and does not assume that Alice’s observations are independent of Bob’s observations—how much information does Eve have for any one realisation?

It seems clear that Eve’s information should always satisfy the following two requirements. Firstly, since Alice and Bob both share their knowledge with Eve, she should have at least as much information as either of them have individually. Secondly, since Eve has less knowledge than Johnny, she should have no more information than Johnny; i.e., in contrast to Indy, Eve should never have more information than Johnny. As the following theorem shows, these two requirements uniquely determine the functional form of Eve’s information:

**Theorem** **1.** *The unique function h(x⊔y) of $p_{X}\left(x\right)$ and $p_{Y}\left(y\right)$ that satisfies h(x,y)≥h(x⊔y)≥h(x),h(y)≥0 for all $p_{XY}\left(x,y\right)$ is*

(21) $$h\left(x⊔y\right)=\max\left(h(x),h(y)\right)\geq 0.$$

**Proof.**  Clearly, the function is lower bounded by $\max\left(h(x),h(y)\right)$. The upper bound is given by the minimum possible h(x,y), which corresponds to the maximum allowed $p_{XY}\left(x,y\right)$. For any $p_{X}\left(x\right)$ and $p_{Y}\left(y\right)$, the maximum allowed $p_{XY}\left(x,y\right)$ is $\min\left(p_{X}\left(x\right),p_{Y}\left(y\right)\right)$, which corresponds to $h\left(x,y\right)=\max\left(h(x),h(y)\right)$.□

Eve’s information is given by the maximum of Alice’s and Bob’s information, or the information content of the most surprising marginal realisation. Although we have defined Eve’s information by requiring it to be no greater than Johnny’s information, it is also clear that Eve also has no more information than Indiana. As such, Eve’s information satisfies the inequality

(22) h(x)+h(y)≥h(x⊔y)≥h(x),h(y)≥0,

which is analogous to the inequality (5) satisfied by measure. Hence, as pre-empted by the notation (and as further justified in Section 6), Eve’s information is referred to as the *union information content*. The union information content is the maximum possible information that Eve can get from knowing what Alice and Bob know—it quantifies the information provided by a joint event (x,y) when one knows the marginal distributions $p_{X}$ and $p_{Y}$, but does not know nor make any assumptions about the joint distribution $p_{XY}$.

Similar to how the conditional information contents (15) and (16) enable us to quantify how much more information Johnny has relative to either Alice or Bob, the inequality (22) enables us to quantify how much information Eve gets from Alice relative to Bob and vice versa, respectively,

(23) $$\begin{array}{cccc} h(x\backslash y) & =h(x⊔y)-h(y) & =\max\left(h(x)-h(y),0\right) & \geq 0, \end{array}$$

(24) $$\begin{array}{cccc} h(y\backslash x) & =h(x⊔y)-h(x) & =\max\left(0,h(y)-h(x)\right) & \geq 0. \end{array}$$

These non-negative functions are analogous to measure on the relative complements of a pair of sets and are called the *unique information content from x relative to y*, and vice versa, respectively. It is easy to see that, since Eve’s information is either equal to Alice’s or Bob’s information (or both), at least one of these two functions must be equal to zero.

The inequality (22) also enables us to quantify how much more information Indiana has relative to Eve. Since Indiana’s assumed information is given by the sum of Alice’s and Bob’s information while Eve’s information is given by the maximum of Alice’s and Bob’s information, the difference between the two is given by the minimum of Alice’s and Bob’s information,

(25) $$h\left(x⊓y\right)=h\left(x\right)+h\left(y\right)-h\left(x⊔y\right)=h\left(x\right)+h\left(y\right)-\max\left(h(x),h(y)\right)=\min\left(h(x),h(y)\right)\geq 0.$$

In contrast to the comparison between Indiana and Johnny, i.e., the mutual information content (18), the comparison between Indiana and Eve is non-negative. As such, this function is analogous to measure on the intersection of two sets and hence will be referred to as the *intersection information content*. The intersection information content is the minimum possible information that Eve could have gotten from knowing either what Alice or Bob know, and is given by the information content of the least surprising marginal realisation.

Finally, from (21) and (23)–(25), it is not difficult to see that Eve’s information can be decomposed into the information that could have been obtained from either Alice or Bob, the unique information from Alice relative to Bob and the unique information from Bob relative to Alice,

(26) h(x⊔y)=h(x⊓y)+h(x\y)+h(y\x).

**Figure 3:**
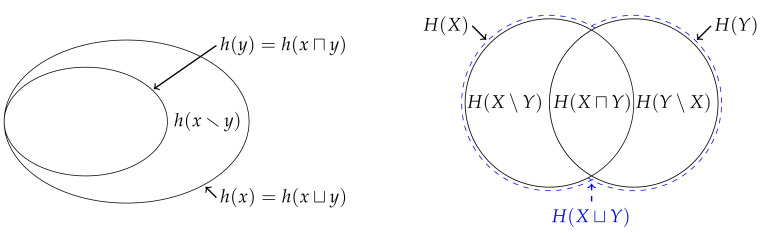
(**Left**) If Alice’s information h(x) is greater than Bob’s information h(y), then Eve’s information h(x⊔y) is equal to Alice’s information h(x). In effect, Eve is pessimistically assuming that information provided by the least surprising marginal realisation h(x⊓y) is already provided by the most surprising marginal realisation h(x⊔y), i.e., Bob’s information h(y) is a subset of Alice’s information h(x). From this perspective, Eve gets unique information from Alice relative to Bob h(x\y), but does not get any unique information from Bob relative to Alice h(y\x)=0. (**Right**) Although for each realisation Eve can only get unique information from either Alice or Bob, it is possible that Eve can expect to get unique information from both Alice and Bob on average. (Do not confuse this representation of the union entropy with the diagram that represents the joint entropy in Figure 1).

Of course, as already discussed, at least one of these unique information contents must be zero. Figure 3 depicts this decomposition for some realisation whereby Alice’s information h(x) is greater than Bob’s information h(y).

To summarise thus far, both Alice and Bob share their information with Indiana and Eve, who then each interpret this information in a different way. By comparing Figure 2 and Figure 3, we can easily contrast their distinct perspectives. Eve is more conservative than Indiana and assumes that she has gotten as little information as she could possibly have gotten from knowing what Alice and Bob know; this is given by the maximum from Alice’s and Bob’s information, or is the information content associated with the most surprising marginal realisation observed by Alice and Bob. In effect, Eve’s conservative approach means that she pessimistically assumes that the information provided by the least surprising marginal realisation was already provided by the most surprising marginal realisation. In contrast, Indiana optimistically assumes that the information provided by the least surprising marginal realisation is independent of the information provided by the most surprising marginal realisation.

Let us now consider the information that Eve expects to get from a single realisation,

(27) $$H\left(X⊔Y\right)=E_{XY}\left[h(x⊔y)\right]\geq 0.$$

This function is called the union entropy, and quantifies the expected surprise of the most surprising realisation from either *X* or *Y*. Similar to how the non-negativity of the entropy (11) follows from the non-negativity of the information content (10), the non-negativity of the union entropy (27) follows directly from the non-negativity of the union information content (21)—i.e., we do not need to invoke Jensen’s inequality. Indeed, the union entropy cannot be written as a Kullback–Leibler divergence.

Since the expectation value is monotonic, and since the union information content satisfies the inequality (22), we get that the union entropy satisfies

(28) H(X)+H(Y)≥H(X⊔Y)≥H(X),H(Y)≥0,

and hence is also analogous to measure on the union of two sets. Using this inequality, we can quantify how much more information Eve expects to get from Alice relative to Bob, or vice versa, respectively,

(29) $$\begin{array}{cccc} H(X\backslash Y) & =H(X⊔Y)-H(Y) & =E_{XY}\left[h\left(x\backslash y\right)\right] & \geq 0, \end{array}$$

(30) $$\begin{array}{cccc} H(Y\backslash X) & =H(X⊔Y)-H(X) & =E_{XY}\left[h\left(y\backslash x\right)\right] & \geq 0, \end{array}$$

These functions are also analogous to measure on the relative complements of a pair of sets and hence will be called the unique entropy from *X* relative to *Y*, and vice versa, respectively. Crucially, and in contrast to (23) and (24), both of these quantities can be simultaneously non-zero; although Alice might observe the most surprising event in one joint realisation, Bob might observe the most surprising event in another and hence both functions can be simultaneously non-zero.

Now, consider how much more information Indiana expects to get relative to Eve,

(31) $$H\left(X⊓Y\right)=H\left(X\right)+H\left(Y\right)-H\left(X⊔Y\right)=E_{XY}\left[h(x⊓y)\right]\geq 0.$$

This function is also analogous to measure on the intersection of two sets function will be called the intersection entropy. In contrast to the mutual information (20), since the intersection information content (25) is non-negative, we do not require an additional proof to show that the intersection entropy is non-negative. Moreover, the intersection entropy cannot be written as a Kullback–Leibler divergence.

Finally, similar to (26), we can decompose Eve’s expected information into the following components,

(32) H(X⊔Y)=H(X⊓Y)+H(X\Y)+H(Y\X).

It is important to reiterate that, in contrast to (26), there is nothing which requires either of the two unique entropies to be zero. Thus, as shown in Figure 3, the Venn diagram which represents the union and intersection entropy differs from that which represents the union information content.

## 4. Synergistic Information Content

As discussed at the beginning of the previous section, and as required in Theorem 1, one of the defining features of Eve’s information is that it is never greater than Johnny’s information,

(33) h(x,y)≥h(x⊔y).

Thus, we can compare how much more information Johnny has relative to Eve,

(34) $$h\left(x⊕y\right)=h\left(x,y\right)-h\left(x⊔y\right)=h\left(x,y\right)-\max\left(h(x),h(y)\right)=\min\left(h(y|x),h(x|y)\right)\geq 0.$$

**Figure 4:**
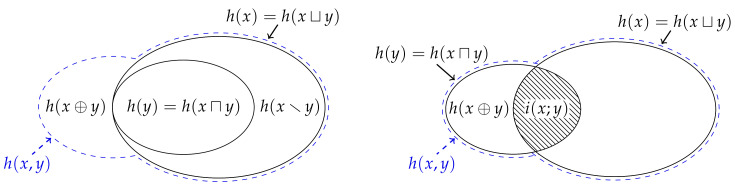
(**Left**) This Venn diagram shows how the synergistic information h(x⊕y) can be defined by comparing the joint information content h(x,y) from Figure 2 to the union information content h(x⊔y) from Figure 3. Note that, for this particular realisation, we are assuming that h(x)>h(y). It also provides a visual representation of the decomposition (40) of the joint information content h(x,y). (**Right**) By rearranging the marginal entropies such that they match Figure 2 (albeit with different sizes here), it is easy to see why the mutual information content i(x;y) is equal to the intersection information content h(x⊓y) minus the synergistic information content h(x⊕y), c.f. (38).

This non-negative function is called the *synergistic information content*, and it quantifies how much more information one gets from knowing the joint probability $p_{XY}\left(x,y\right)$ relative to merely knowing the marginal probabilities $p_{X}\left(x\right)$ and $p_{Y}\left(y\right)$. Figure 4 shows how this relationship can represented using a Venn diagram. Of course, by this definition, Johnny’s information is equal to the union information content plus the synergistic information content, and hence, by using (26), we can decompose Johnny’s information into the intersection information content, the unique information contents and the synergistic information contents,

(35) h(x,y)=h(x⊔y)+h(x⊕y)=h(x⊓y)+h(x\y)+h(y\x)+h(x⊕y).

This decomposition can be seen in Figure 4, although it is important to recall that at least one of h(x\y) and h(y\x) must be equal to zero. In a similar manner, the extra information that Johnny has relative to Bob (13) can be decomposed into the unique information content from Alice and the synergistic information content, and vice versa for the extra information that Johnny has relative to Alice (14),

(36) $$\begin{array}{cc} h(x|y) & =h(x\backslash y)+h(x⊕y), \end{array}$$

(37) $$\begin{array}{cc} h(y|x) & =h(y\backslash x)+h(x⊕y). \end{array}$$

Now, recall that the mutual information content (18) is given by Indiana’s information minus Johnny’s information. By replacing Johnny’s information with the union information content plus the synergistic information content via (34) and rearranging using (25), we get that the mutual information content is equal to the intersection information content minus the synergistic information content,

(38) $$\begin{array}{c} i(x;y)=h(x)+h(y)-h(x,y)=h(x)+h(y)-h(x⊔y)-h(x⊕y)=h(x⊓y)-h(x⊕y). \end{array}$$

Indeed, this relationship can be identified in Figure 4. Clearly, the mutual information content is negative whenever the synergistic information content is greater than the intersection information content. From this perspective, the mutual information content can be negative because there is nothing to suggest that the synergistic information content should be no greater than the intersection information content. In other words, the additional surprise associated with knowing $p_{XY}\left(x,y\right)$ relative to merely knowing $p_{X}\left(x\right)$ and $p_{Y}\left(y\right)$ can exceed the surprise of the least surprising marginal realisation.

Let us now quantify how much more information Johnny expects to get relative to Eve,

(39) $$H\left(X⊕Y\right)=E_{XY}\left[h(x⊕y)\right]=H\left(X,Y\right)-H\left(X⊔Y\right)\geq 0,$$

which we call the synergistic entropy. Crucially, although the synergistic information content is given by the minimum of the two conditional information contents, the synergistic entropy does not in general equal one of the two the conditional entropies. This is because, although Alice might observe the most surprising event in one joint realisation such that the synergistic information content is equal to Bob’s information given Alice’s information, Bob might observe the most surprising event in another realisation such that the synergistic information content is equal to Alice’s information given Bob’s information for that particular realisation. Thus, the synergistic entropy does not equal the conditional entropy for the same reason that unique entropies (29) and (30) can be simultaneously non-zero.

With the definition of synergistic entropy, it is not difficult to show that, similar to (35), the joint entropy can be decomposed into the following components,

(40) H(X,Y)=H(X⊔Y)+H(X⊕Y)=H(X⊓Y)+H(X\Y)+H(Y\X)+H(X⊕Y).

**Figure 5:**
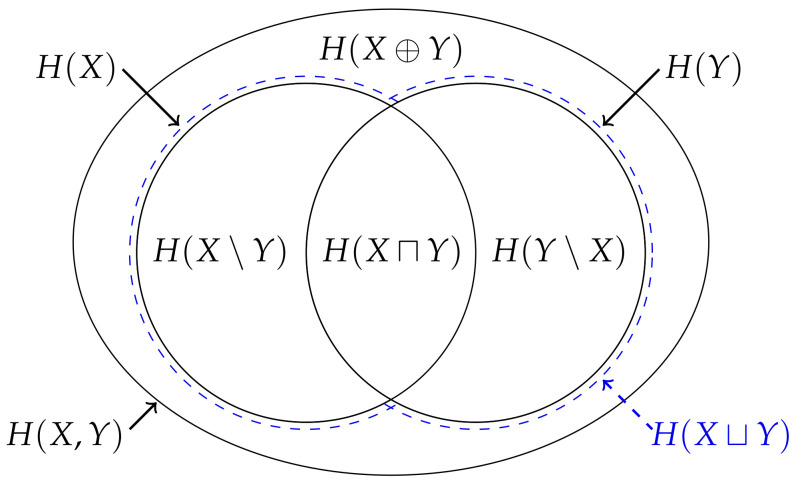
This Venn diagram shows how the synergistic entropy H(X⊕Y) can be defined by comparing the joint entropy H(X,Y) from Figure 1 to the union entropy H(X⊔Y) from Figure 3. It also provides a visual representation of the decomposition (40) of the joint entropy H(X,Y).

Figure 5 depicts this decomposition using a Venn diagram, and shows how the union entropy from Figure 3 is related to the joint entropy H(X,Y). Likewise, similar to (36) and (37), it is easy to see that conditional entropies can be decomposed as follows,

(41) $$\begin{array}{cc} H(X|Y) & =H(X\backslash Y)+H(X⊕Y), \end{array}$$

(42) $$\begin{array}{cc} H(Y|X) & =H(Y\backslash X)+H(X⊕Y) \end{array}$$

Finally, as with (38), we can also show that the mutual information is equal to the intersection entropy minus the synergistic entropy,

(43) I(X;Y)=H(X⊓Y)−H(X⊕Y)≥0.

Although there is nothing to suggest that the synergistic information content must be no greater than the intersection information content, we know that the synergistic entropy must be no greater than the intersection entropy because I(X;Y)≥0. In other words, the expected difference between the surprise of the joint realisation and the most surprising marginal realisation cannot exceed the expected surprise of the least surprising realisation.

## 5. Properties of the Union and Intersection Information Content

Theorem 1 determined the function form of Eve’s information when Alice and Bob share their knowledge with her. We now wish to generalise this result to consider the situation whereby an arbitrary number of marginal observers share their information with Eve. Rather than try to directly determine the functional form, however, we proceed by considering the algebraic structure of shared marginal information.

If Alice and Bob observe the same realisation *x* such that they have the same information h(x), then upon sharing we would intuitively expect Eve to have the same information h(x). Similarly, the minimum information that Eve could have received from either Alice or Bob should be the same information h(x). Since the maximum and minimum operators are idempotent, the union and intersection information content both align with this intuition.

**Property** **1** (Idempotence)**.** *The union and intersection information content are idempotent,*

(44) $$\begin{array}{cc} h(x⊔x) & =h(x), \end{array}$$

(45) $$\begin{array}{cc} h(x⊓x) & =h(x). \end{array}$$

It also seems reasonable to expect that Eve’s information should not depend on the order in which Alice and Bob share their information, nor should the minimum information that Eve could have received from either individual. Again, since the maximum and minimum operators are commutative, the union and intersection information content both align with our intuition.

**Property** **2** (Commutativity)**.** *The union and intersection information content are commutative,*

(46) $$\begin{array}{cc} h(x⊔y) & =h(y⊔x), \end{array}$$

(47) $$\begin{array}{cc} h(x⊓y) & =h(y⊓x). \end{array}$$

Now, suppose that Charlie is another individual who, similar to Alice and Bob, is separately observing some process, and let the random variable *Z* represent her observations. Say that Dan is yet another individual with whom, similar to Eve, our observers can share their information. Intuitively, it should not matter whether Alice, Bob and Charlie share their information directly with Eve, or whether they share their information through Dan. To be specific, Alice and Bob could share their information with Dan such that his information is given by h(x⊔y), and then Charlie and Dan could subsequently share their information with Eve such that her information is given by $h\left((x⊔y)⊔z\right)$. Similarly, Bob and Charlie could share their information with Dan such that his information is given by h(y⊔z), and then Alice and Dan could subsequently share their information with Eve such that her information is given by $h\left(x⊔(y⊔z)\right)$. Alternatively, Alice, Bob and Charlie could entirely bypass Dan and share their information directly with Eve such that her information is given by h(x⊔y⊔z). Since the maximum operator is associative, the union information content is the same in all three cases and hence aligns with our intuition. A similar argument can be made to show that the intersection information content is also associative.

**Property** **3** (Associative)**.** *The union and intersection information content are associative,*

(48) $$\begin{array}{ccc} h(x⊔y⊔z) & =h\left((x⊔y)⊔z\right) & =h\left(x⊔(y⊔z)\right), \end{array}$$

(49) $$\begin{array}{ccc} h(x⊓y⊓z) & =h\left((x⊓y)⊓z\right) & =h\left(x⊓(y⊓z)\right), \end{array}$$

Suppose now that Alice and Bob share their information with Dan such the information that he could have gotten from either Alice or Bob is given by h(x⊓y). If Alice and Dan both share their information with Eve, then Eve’s information is given by

(50) $$h\left(x⊔(x⊓y)\right)=\max\left(h\left(x\right),\min\left(h(x),h(y)\right)\right)=h\left(x\right),$$

and hence Bob’s information has been absorbed by Alice’s information. Now, suppose that Alice and Bob share their information with Dan such his information is given by h(x⊔y). If Alice and Dan both share their information with Eve, then the information that Eve could have gotten from either Alice or Dan is given by

(51) $$h\left(x⊓(x⊔y)\right)=\min\left(h\left(x\right),\max\left(h(x),h(y)\right)\right)=h\left(x\right).$$

Again, Bob’s information has been absorbed by Alice’s information. Both of these results are a consequence of the fact that the maximum and minimum operators are connected to each other by the absorption identity.

**Property** **4** (Absorption)**.** *The union and intersection information content are connected by absorption,*

(52) $$\begin{array}{cc} h\left(x⊔(x⊓y)\right) & =h(x), \end{array}$$

(53) $$\begin{array}{cc} h\left(x⊓(x⊔y)\right) & =h(x). \end{array}$$

Now, say that Daniella is, similar to Eve or Dan, an individual with whom our observers can share their information. Consider the following two cases: Firstly, suppose that Bob and Charlie share their information with Dan such that the information that Dan could have gotten from either Bob or Charlie is given by h(y⊓z). If both Alice and Dan share their information with Eve, then her information is given by $h\left(x⊔(y⊓z)\right)$. In the second case, suppose that Alice and Bob share their information with Dan such that his information is given by h(x⊔y), while Alice and Charlie simultaneously share their information with Daniella such that her information is given by h(x⊔z). If Dan and Daniella both share their information with Eve, then the information that she could have gotten from either Dan or Daniella is then given by $h\left((x⊔y)⊓(x⊔z)\right)$. In both cases, Eve has the same information since the maximum operator is distributive,

(54) $$\begin{array}{cc} h\left(x⊔(y⊓z)\right) & =\max\left(h\left(x\right),\min\left(h(y),h(z)\right)\right)\\ \; & =\min\left(\max\left(h(x),h(y)\right),\max\left(h(x),h(z)\right)\right)=h\left((x⊔y)⊓(x⊔z)\right). \end{array}$$

Since the maximum and minimum operators are distributive over each other, regardless of whether Eve gets Alice’s information and Bob’s or Charlie’s information, or if Eve gets Alice’s and Bob’s information or Alice’s and Charlie’s information, Eve has the same information. The same reasoning can be applied to show that, regardless of whether Eve gets Alice’s information or Bob’s and Charlie’s information, or if Eve gets Alice’s or Bob’s information and Alice’s or Charlie’s information, Eve has the same information.

**Property** **5** (Distributivity)**.** *The union and intersection information content are distribute over each other,*

(55) $$\begin{array}{cc} h\left(x⊔(y⊓z)\right) & =h\left((x⊔y)⊓(x⊔z)\right), \end{array}$$

(56) $$\begin{array}{cc} h\left(x⊓(y⊔z)\right) & =h\left((x⊓y)⊔(x⊓z)\right). \end{array}$$

Now, consider a set of *n* individuals and let $X=\{X_{1},X_{2},\ldots ,X_{n}\}$ be the joint random variable that represents their observations. Suppose that these individuals together observe the joint realisation $x=\{x_{1},x_{2},\ldots ,x_{n}\}$ from X. By Property 3 and the general associativity theorem, it is clear that Eve’s information is given by

(57) $$h\left(x_{1}⊔x_{2}⊔\ldots ⊔x_{n}\right)=\max\left(h\left(x_{1}\right),h\left(x_{2}\right),\ldots ,h\left(x_{n}\right)\right)\geq 0,$$

while the minimum information that Eve could have gotten from any individual observer is given by

(58) $$h\left(x_{1}⊓x_{2}⊓\ldots ⊓x_{n}\right)=\min\left(h\left(x_{1}\right),h\left(x_{2}\right),\ldots ,h\left(x_{n}\right)\right)\geq 0.$$

This accounts for the situation whereby *n* marginal observers directly share their information with Eve, and could clearly be considered for any subset S of the observers x. We now wish to consider all of the distinct ways that these marginal observers can share their information indirectly with Eve. As the following theorem shows, Properties 1–5 completely characterise the unique methods of marginal information sharing.

**Theorem** **2.** *The marginal information contents form a join semi-lattices 〈x,h(⊔)〉 under the *max* operator. Separately, the marginal information contents form a meet semi-lattice 〈x,h(⊓)〉 under the *min* operator.*

**Proof.**  Properties 1–3 completely characterise semi-lattices [16,17]. □

**Theorem** **3.** *The marginal information contents form a distributive lattice 〈x,h(⊔),h(⊓)〉 under the* max *and* min *operators.*

**Proof.**  From Property 4, we have that the semi-lattices 〈x,h(⊔)〉 and 〈x,h(⊓)〉 are connected by absorption and hence form a lattice 〈x,h(⊔),h(⊓)〉. By Property 5, this is a distributive lattice [16,17].□

**Figure 6:**
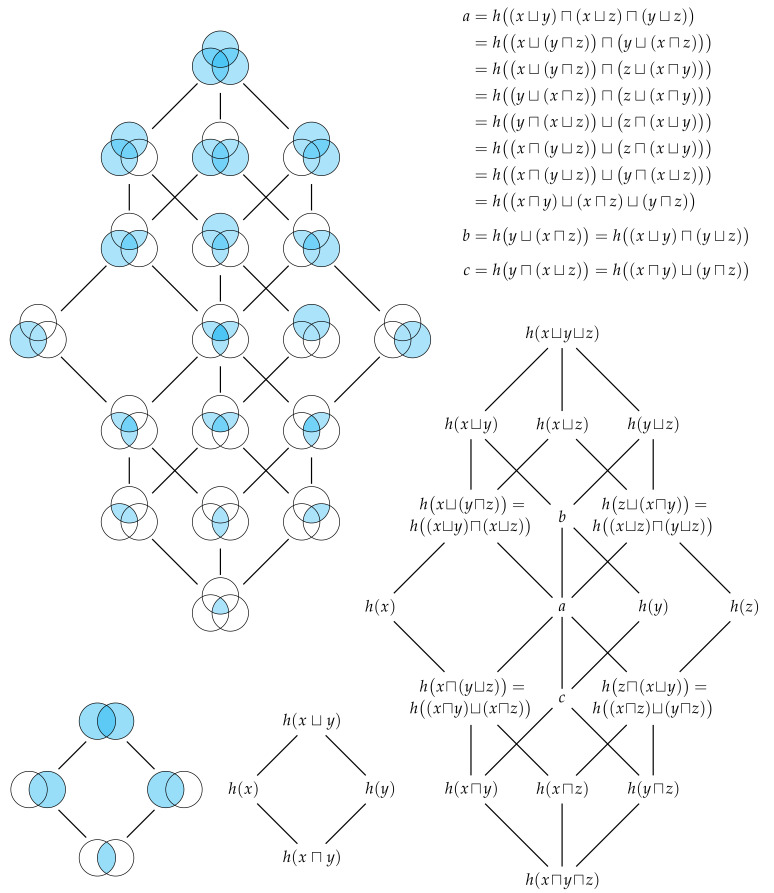
(**Bottom right**) The distributive lattices 〈x,h(⊔),h(⊓)〉 of information contents for two and three and three observers. It is also important to note that, by replacing *h*, *x*, *y* and *z* with *H*, *X*, *Y* and *Z*, respectively, we can obtain the distributive lattices for entropy. In fact, this is crucial since Property 6 enables us to reduce the distributive lattice of information contents to a mere total order; however, this property does not apply to the entropies, and hence we cannot further simplify the lattice of entropies. (**Top left**) By the fundamental theorem of distributive lattices, the distributive lattices of marginal information contents has a one-to-one correspondence with the lattice of sets. Notice that the lattice for two sets corresponds to the Venn diagram for entropies in Figure 3.

Each way that a set of *n* observers can share their information with Eve such that she has distinct information corresponds to an element in partially ordered set, or more specifically the free distributive lattice on *n* generators [16]. Figure 6 shows the free distributive lattices generated by n=2 and n=3 observers. The number of elements in this lattice is given by the (*n*)th Dedekind number (p. 273 [18]) (see also [19]). By the fundamental theorem of distributive lattices (or Birkhoff’s representation theory), there is isomorphism between the union information content and set union, and between the intersection information content and set intersection [16,17,20,21]. It is this one-to-one correspondence that justifies our use of the terms union and intersection information content for *n* variables in general. Every identity that holds in a lattice of sets will have a corresponding identity in this distributive lattice of information contents. Figure 6 also depicts the sets which correspond to each term in the lattice of information contents. Just as the cardinality of sets is non-decreasing as we consider moving up through the various terms in a lattice of sets, Eve’s information is non-decreasing as we moving up through the various terms in the corresponding lattice of information contents. In particular, we can quantify the unique information content that Eve gets from one method of information sharing relative to any other method that is lower in the lattice.

Every property of the union and intersection information content that we have considered thus far has been directly inherited by the union and intersection entropy. However, there is one final property is not inherited by the entropies. If Alice and Bob share their information with Eve, then Eve’s information is given by either Alice’s or Bob’s information, and similar for the information that Eve could have gotten from either Alice or Bob. As the subsequent theorem shows, this property enables us to greatly reduce the number of distinct terms in the distributive lattice for information content since any partially ordered set with a connex relation forms a total order.

**Property** **6** (Connexity)**.** *The union and intersection information content are given by at least one of*

(59) h(x⊔y)=h(x)andh(x⊓y)=h(y),orh(x⊔y)=h(y)andh(x⊓y)=h(x)

## 6. Generalised Marginal Information Sharing

We now use Properties 1–6 to generalise the results of Theorem 1 and Section 3.

**Theorem** **4.** *The marginal information contents are a totally ordered set under the max and min operators.*

**Proof.**  A totally ordered set is a partially ordered set with the connex property (p. 2 [16]).□

**Figure 7:**
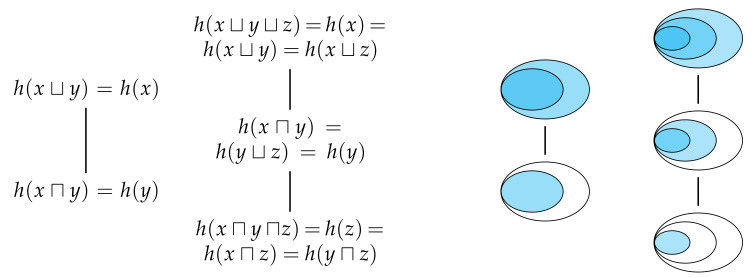
(**Left**) The total order of marginal information contents for two and three observers, whereby we have assumed that Alice’s information h(x) is greater than Bob’s information h(y), which is greater than Charlie’s information h(z). It is important to note that taking the expectation value over these information contents for each realisation, which may each have a different total orders, yields entropies which are merely partially ordered. It is for this reason that Property 6 does not apply to entropies. (**Right**) The Venn diagrams corresponding to the total order for for two and three observers and their corresponding information contents. Notice that the total order for two sets corresponds to the Venn diagram for information contents in Figure 3.

Figure 7 shows the totally ordered sets generated by n=2 and n=3 observers, and also depicts the corresponding sets. Although the number of distinct terms has been reduced, Eve’s information is still non-decreasing as we move up through terms of the totally ordered set. If we now compare how much unique information Eve gets from a given method of information sharing relative to any other method of information sharing which is equal or lower in the totally ordered set, then we obtain a result which generalises (23) and (24) to consider more than two observers. Similarly, this total order enables us to generalise (25) using the maximum–minimum identity [22], which is a form of the principle of inclusion–exclusion [21] for a totally ordered set,

(60) $$\begin{array}{cc} h(x_{1}⊓x_{2}⊓\ldots ⊓x_{n})\; & =\min\left(h\left(x_{1}\right),h\left(x_{2}\right),\ldots ,h\left(x_{n}\right)\right)\\ \; & =\sum_{k=1}^{n}{\left(-1\right)}^{k-1}\sum_{\begin{array}{c} S\subseteq x\\ |S|=k \end{array}}\max\left(h\left(s_{1}\right),h\left(s_{2}\right),\ldots ,h\left(s_{k}\right)\right)\\ \; & =\sum_{k=1}^{n}{\left(-1\right)}^{k-1}\sum_{\begin{array}{c} S\subseteq x\\ |S|=k \end{array}}h\left(s_{1}⊔s_{2}⊔\ldots ⊔s_{k}\right), \end{array}$$

or, conversely,

(61) $$h\left(x_{1}⊔x_{2}⊔\ldots ⊔x_{n}\right)=\sum_{k=1}^{n}{\left(-1\right)}^{k-1}\sum_{\begin{array}{c} S\subseteq x\\ |S|=k \end{array}}h\left(s_{1}⊓s_{2}⊓\ldots ⊓s_{k}\right).$$

Now that we have generalised the union and intersection information content, similar to Section 3, let us now consider taking the expectation value for each term in the distributive lattice. For every joint realisation x from X, there is a corresponding distributive lattice of information contents. Hence, similar to (27) and (29)–(31), we can consider taking the expectation value of each term in the lattice over all realisations. Since the expectation is a linear operator, this yields a set of entropies that are also idempotent, commutative, associative, absorptive and distributive, only now over the random variables from X. Thus, the information that Eve expects to gain from a single realisation for a particular method of information sharing also corresponds to a term in a free distributive lattice generated by *n*. This distributive lattice for entropies can be seen in Figure 6 by replacing *x*, *y*, *z* and *h* with *X*, *Y*, *Z* and *H*, respectively.

Crucially, however, Property 6 does not hold for the entropies—it is not true that Eve’s expected information H(X⊔Y) is given by either Alice’s expected information H(X) or Bob’s expected information H(Y). Thus, despite the fact that the distributive lattice of information content can be reduced to a total order, the distributive lattice of entropies remains partially ordered. Although the information contents are totally ordered for every realisation, this order is not in general the same for every realisation. Consequently, when taking the expectation value across many realisations to yield the corresponding entropies, the total order is not maintained, and hence we are left with a partially ordered set of entropies. Indeed, we already saw the consequences of this result in Figure 3 whereby Alice’s and Bob’s information content was totally ordered for any one realisation, but their expected information was partially ordered.

## 7. Multivariate Information Decomposition

In Section 4, we use the shared marginal information from Section 3 to decompose the joint information content into four distinct components. Our aim now is to use the generalised notion of shared information from the previous section to produce a generalised decomposition of the joint information content. To begin, suppose that Johnny observes the joint realisation (x,y,z) while Alice, Bob and Charlie observe the marginal realisations *x*, *y* and *z*, respectively, and say that Alice, Bob and Charlie share their information with Eve such that her information is given by h(x⊔y⊔z). Clearly, Johnny has at least as much information as Eve,

(62) h(x,y,z)≥h(x⊔y⊔z).

Thus, we can compare how much more information Johnny has relative to Eve,

(63) $$h\left(x⊕y⊕z\right)=h\left(x,y,z\right)-h\left(x⊔y⊔z\right)=\min\left(h(y,z|x),h(x,z|y),h(x,y|z)\right)\geq 0.$$

**Figure 8:**
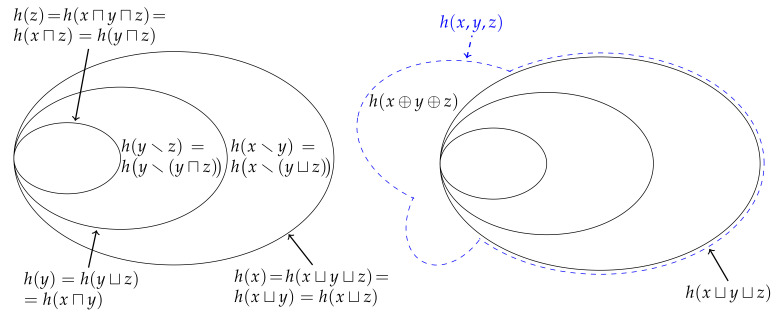
Similar to Figure 4, this Venn diagram shows how the synergistic information h(x⊕y⊕z) can be defined by comparing the joint information content h(x,y,z) to the union information content h(x⊔y⊔z). Note that, for this particular realisation, we are assuming that h(x)>h(y)>h(z).

This non-negative function generalises the earlier definition of the synergistic information content (34) such that it now quantifies how much information one gets from knowing the joint probability $p_{XYZ}\left(x,y,z\right)$ relative to merely knowing the three marginal probabilities $p_{X}\left(x\right)$, $p_{Y}\left(y\right)$ and $p_{Z}\left(z\right)$. Figure 8 shows how this relationship can be represented using a Venn diagram.

Now, consider three more observers, Joan, Jonas, and Joanna, who observe the joint marginal realisations (x,y), (x,z) and (y,z), respectively. Clearly, these additional observers greatly increase the number of distinct ways in which marginal information might be shared with Eve. For example, if Alice and Joanna share their information, then Eve’s information is given by $h\left(x⊔(y,z)\right)$. Alternatively, if Joan and Jonas share their information, then Eve’s information is given by $h\left((x,y)⊔(x,z)\right)$. Perhaps most interestingly, if Joan, Jonas and Joanna share their information, then Eve’s information is given by $h\left((x,y)⊔(x,z)⊔(y,z)\right)$. Moreover, we know that Johnny has at least as much information as Eve has in this situation,

(64) $$h\left(x,y,z\right)\geq h\left((x,y)⊔(x,z)⊔(y,z)\right).$$

Thus, by comparing how much more information Johnny has relative to Eve in this situation, we can define a new type of synergistic information content that quantifies how much information one gets from knowing the full joint realisation to merely knowing all of the pairwise marginal realisations,

(65) $$h\left((x,y)⊕(x,z)⊕(y,z)\right)=h\left(x,y,z\right)-h\left((x,y)⊔(x,z)⊔(y,z)\right)=\min\left(h(z|x,y),h(y|x,z),h(x|y,z)\right).$$

Of course, these new ways to share joint information are not just restricted to the union information. If Alice and Joanna share their information, then the information that Eve could have gotten from either is given by $h\left(x⊓(y,z)\right)$. It is also worthwhile noting that this quantity is not less than the information that Eve could have gotten from either Alice’s information or Bob’s and Charlie’s information,

(66) $$h\left(x⊓(y,z)\right)\geq h\left(x⊓(y⊔z)\right).$$

Thus, we can also consider defining new types of synergistic information content associated with these this mixed type comparisons,

(67) $$h\left(x⊓(y⊕z)\right)=h\left(x⊓(y,z)\right)-h\left(x⊓(y⊔z)\right).$$

However, it is important to note that this quantity does not equal $\min\left(h(x),\min(h(z|y),h(y|z))\right)$.

**Figure 9:**
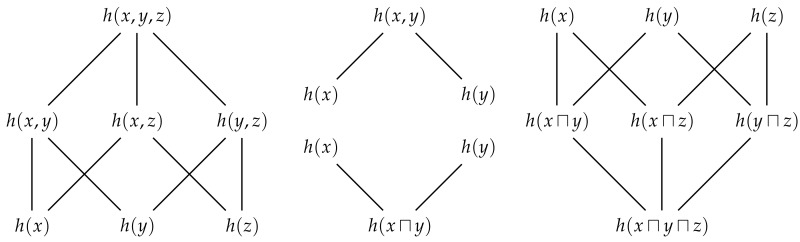
(**Top-middle and left**) The join semi-lattice $\langle h\;;(\;,)\rangle$ for n=2 and n=3 marginal observers. Johnny’s information is always given by the joint information content at the top of the semi-lattice, while the information content of individuals such as Alice, Bob and Charlie who observe single realisations are found at the bottom of the semi-lattice. The information content of joint marginal observers such as Joanna, Jonas and Joan are found in between these two extremities. (**Bottom-middle and right**) The meet semi-lattice $\langle h\;;⊓\rangle$ for n=2 and n=3 marginal observers. Since these two semi-lattices are not connect by absorption, their combined structure is not a lattice.

With all of these new ways to share joint marginal information, it is not immediately clear how we should decompose Johnny’s information. Nevertheless, let us begin by considering the algebraic structure of joint information content. From the inequality (12), we know that any pair of marginal information contents h(x) and h(y) are upper-bounded by the joint information content h(x,y). It is also easy to see that the joint information content is idempotent, commutative and associative. Together, these properties are sufficient for establishing that the algebraic structure of joint information content is that of a join semi-lattice [16] which we denote by 〈x;h(,)〉. Figure 9 shows the semi-lattices generated by n=2 and n=3 observers.

We now wish to establish the relationship between this semi-lattice of joint information content 〈x;h(,)〉 and the distributive lattice of shared marginal information 〈x;h(⊔),h(⊓)〉. In particular, since our aim is to decompose Johnny’s information, consider the relationship between the join semi-lattice 〈x;h(,)〉 and the meet semi-lattice 〈x;h(⊓)〉, which is also depicted in Figure 9. In contrast to the semi-lattice of union information content 〈x;h(⊔)〉, the semi-lattice 〈x;h(,)〉 is not connected to the semi-lattice 〈x;h(⊓)〉. Although the intersection information content absorbs the joint information content, since

(68) $$h\left(x⊓(x,y)\right)=h\left(x\right)$$

for all h(x) and h(y), the joint information content does not absorb the intersection information content since $h\left(x,(x⊓y)\right)$ is equal to h(x,y) for h(x)≥h(y), i.e., is not equal to h(x) as required for absorption. Since the the join semi-lattice 〈x;h(,)〉 is not connected to the meet semi-lattice 〈x;h(⊓)〉 by absorption, their combined algebraic structure is not a lattice.

Despite the fact that the overall algebraic structure is not a lattice, there is a lattice sub-structure 〈A(x),⪯〉 within the general structure. This substructure is isomorphic to the redundancy lattice from the partial information decomposition [23] (see also [24]), and its existence is a consequence of the fact that the intersection information content absorbs the joint information content in (68). To identify this lattice, we must first determine the reduced set of elements A(x) upon which it is defined. We begin by considering the set of all possible joint realisations which is given by $P_{1}\left(x\right)$ where $P_{1}\left(x\right)=P\left(x\right)\backslash ⊘$. Elements of this set $P_{1}\left(x\right)$ correspond to the elements from the join semi-lattice 〈x;h(,)〉, e.g., the elements {x} and {x,y} correspond to h(x) and h(x,y), respectively. In alignment with Williams and Beer [23], we call the elements of $P_{1}\left(x\right)$ *sources* and denote them by $A_{1},A_{2},\ldots ,A_{k}$. Next, we consider set of all possible *collections of sources* which are given by the set $P_{1}\left(P_{1}\left(x\right)\right)$. Each collection of sources corresponds to an element of the meet semi-lattice $\langle P_{1}\left(x\right);h\left(\;⊓\;\right)\rangle$, or a particular way in which we can evaluate the intersection information content of a group of joint information contents. For example, the collections of sources {{x},{y}} and {{x},{y,z}} correspond to the h(x⊓y) and $h\left(x⊓(y,z)\right))$, respectively. Not all of these collections of sources are distinct, however. Since the intersection information content absorbs the joint information content, we can remove the element {{x},{x,y}} corresponding to $h\left(x⊓(x,y)\right)$ as this information is already captured by the element {{x}} corresponding to h(x). In general, we can remove any collection of sources that corresponds to the intersection information content between a source $A_{i}$ and any source $A_{j}$ that is in the down-set $↓\;A_{i}$ with respect to the join semi-lattice $\langle x;h(\;,)\rangle$. (A definition of the down-set can be found in [17]. Informally, the down-set ↓A is the set of all elements that precede A.) By removing all such collections of sources, we get the following reduced set of collections of sources,

(69) $$A\left(x\right)=\{\alpha \in P_{1}\left(P_{1}\left(x\right)\right):\forall \;A_{i},\;A_{j}\in \alpha ,\;A_{i}⊄A_{j}\}.$$

Formally, this set corresponds to the set of antichains on the lattice $\langle P_{1}\left(x\right),\subseteq \rangle$, excluding the empty set [23].

Now that we have determined the elements upon which the lattice sub-structure is defined, we must show that they indeed form a lattice. Recall that when constructing the set A(x), we first considered the ordered elements of the semi-lattice $\langle x;h(\;,)\rangle$ and then subsequently consider the ordered elements of the semi-lattice $\langle P_{1}\left(x\right);h\left(\;⊓\;\right)\rangle$. Thus, we need to show that these two orders can be combined together into one new ordering relation over the set A(x). This can be done by extending the approach underlying the construction of the set A(x) to consider any pair of collections of sets α and β from A(x). In particular, the collection of sets β precedes the collection of sets α if and only if for every source B from β, there exists a source A from α such that A is in the down-set ↓B with respect to the join-semi-lattice $\langle x;h(\;,)\rangle$, or formally,

(70) ∀α,β∈A(x),(α⪯β⇔∀B∈β,∃A∈α,A⊆B).

**Figure 10:**
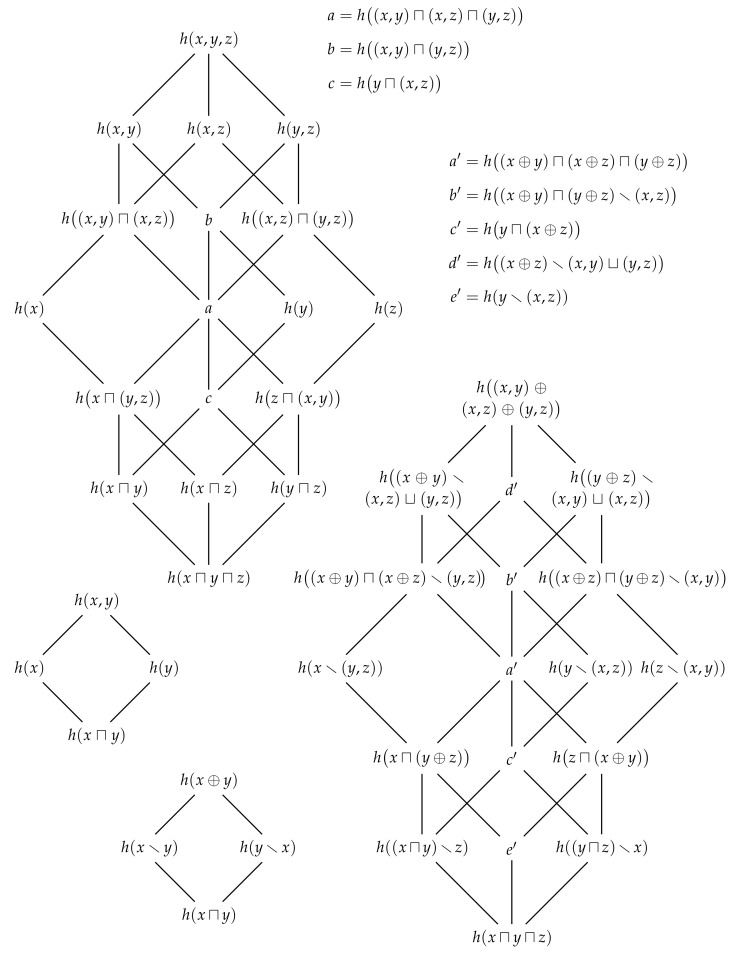
(**Top left**) The redundancy lattices 〈A(x),⪯〉 of information contents for two and three and three observers. Each note in the lattice corresponds to an element in A(x) from (69), while the ordering between elements is given by ⪯ from (70). (**Bottom right**) The partial information contents $h_{\partial }\left(\alpha \right)$ corresponding to the redundancy lattices of information contents for two and three observers.

The fact that 〈A(x),⪯〉 forms a lattice was proved by Crampton and Loizou [25,26] where the corresponding lattice is denoted $\langle A\left(X\right),{⪯}^{\prime }\rangle$ in their notation. Furthermore, they showed that this lattice is isomorphic to the distributive lattices, and hence the number of elements in the set A(x) for *n* marginal observers is also given by the (*n*)th Dedekind number (p. 273 [18]) (see also [19]). Crampton and Loizou [26] also provided the meet ∧ and join ∨ operations for this lattice, which are given by

(71) $$\begin{array}{cc} \alpha \wedge \beta & =\underline{\alpha ⊔\beta }, \end{array}$$

(72) $$\begin{array}{cc} \alpha \vee \beta & =\underline{↑\;\alpha \cap ↑\;\beta }, \end{array}$$

where $\underline{\alpha }$ denotes the set of minimal elements of α with respect to the semi-lattice $\langle x;h(\;,)\rangle$. (A definition of the set of minimal elements can be found in [17]. Informally, $\underline{\alpha }$ is the set of sources of α that are not preceded by any other sources from α with respect to the semi-lattice $\langle x;h(\;,)\rangle$.) This lattice 〈A(x),⪯〉 is the aforementioned sub-structure that is isomorphic to the *redundancy lattice* from Williams and Beer [23]. However, as it is a lattice over information contents, it is actually equivalent to the specificity lattice from [27]. Figure 10 depicts the redundancy lattice of information contents for n=2 and n=3 marginal observers.

Similar to how Eve’s information is non-decreasing as we move up through the terms of the distributive lattice of shared information, the redundancy lattice of information contents enables to see that, for example, the information that Eve could have gotten from either Alice or Joanna $h\left(x⊓(y,z)\right)$ is no less than the information that Eve could have gotten from Alice or Bob h(x⊓y). Thus, by taking the information h(α) associated with the collection of sources α from A(x) and subtracting from it the information $h(\alpha_{i})$ associated with any collection of sources $\alpha_{i}$ from the down-set ↓α, we can evaluate the unique information $h(\alpha \backslash \alpha_{i})$ provided by α relative to $\alpha_{i}$. Moreover, as per Williams and Beer [23], we can derive a function that quantifies the *partial information content* $h_{\partial }\left(\alpha \right)$ associated with the collection of sources α that is not available in any of the collections of sources that are covered by α. (The set of collections of sources that are covered by α is denoted $\alpha^{-}$. A definition of the covering relation is provided in [17]. Informally, $\alpha^{-}$ is the set collections of sources that immediately precede α.) Formally, this function corresponds to the Möbius inverse of *h* on the redundancy lattice 〈A(x),⪯〉, and can be defined implicitly by

(73) $$h\left(\alpha \right)=\sum_{\beta ⪯\alpha }h_{\partial }\left(\beta \right).$$

By subtracting away the partial information terms that strictly precede α from both sides, it is easy to see that the partial information content $h_{\partial }\left(\alpha \right)$ can be calculated recursively from the bottom of the redundancy lattice of information contents,

(74) $$h_{\partial }\left(\alpha \right)=h\left(\alpha \right)-\sum_{\beta ≺\alpha }h_{\partial }\left(\beta \right),$$

As the following theorem shows, the partial information content $h_{\partial }\left(\alpha \right)$ can be written in closed-form.

**Theorem** **5.** *The partial information content $h_{\partial }\left(\alpha \right)$ is given by*

(75) $$\begin{array}{cc} h_{\partial }\left(\alpha \right)\; & =h\left(\alpha \right)-h\left(\alpha_{1}^{-}⊔\alpha_{2}^{-}⊔\ldots ⊔\alpha_{|\alpha^{-}|}^{-}\right)\\ \; & =h\left(\alpha \right)-\max\left(h\left(\alpha_{1}^{-}\right),h\left(\alpha_{2}^{-}\right),\ldots ,h\left(\alpha_{|\alpha^{-}|}^{-}\right)\right)\geq 0, \end{array}$$

*where each $\alpha_{i}^{-}$ is a collection of sets from $\alpha^{-}$.*

**Proof.**  For S⊆A(x), define the set-additive function

(76) $$f\left(S\right)=\sum_{\beta \in S}h_{\partial }\left(\beta \right).$$

From (73), we have that h(α)=f(↓α). The partial information can then by subtracting the set additive on the down-set ↓α from the set additive function on the strict down-set $\dot{↓}\alpha$,

(77) $$h_{\partial }\left(\alpha \right)=f\left(↓\;\alpha \right)-f\left(\dot{↓}\;\alpha \right)=f\left(↓\;\alpha \right)-f\left(\underset{\beta \in \alpha^{-}}{⋃}↓\;\beta \right),$$

By applying the principle of inclusion-exclusion [21], we get that

(78) $$h_{\partial }\left(\alpha \right)=f\left(↓\;\alpha \right)-\sum_{k=1}^{|\alpha^{-}|}{\left(-1\right)}^{k-1}\sum_{\begin{array}{c} S\subseteq \alpha^{-}\\ |S|=k \end{array}}f\left(\underset{\sigma \in S}{⋂}↓\;\sigma \right).$$

For any lattice *L* and A⊆L, we have that ${⋂}_{a\in A}↓\;a$ is equal to ↓(⋀A) (p. 57 [17]), and since the meet operation is given by the intersection information content, we have that

(79) $$\begin{array}{cc} h_{\partial }\left(\alpha \right) & =f\left(↓\;\alpha \right)-\sum_{k=1}^{|\alpha^{-}|}{\left(-1\right)}^{k-1}\sum_{\begin{array}{c} S\subseteq \alpha^{-}\\ |S|=k \end{array}}h\left(s_{1}⊓s_{2}⊓\ldots ⊓s_{k}\right)\\ \; & =h\left(\alpha \right)-h\left(\alpha_{1}⊔\alpha_{2}⊔\ldots ⊔\alpha_{|\alpha^{-}|}\right), \end{array}$$

where the final step has been made using (61) and (76).□

The closed-form solution (75) from Theorem 5 is the same as the closed-form solution presented in Theorem A2 from Finn and Lizier [27]. This, together with the aforementioned fact that the lattice 〈A(x),⪯〉 is equivalent to the specificity lattice, means that each partial information content $h_{\partial }\left(\alpha \right)$ is equal to the partial specificity $i_{\partial }^{+}\left(\alpha \to t\right)$ from (A22) of [27]. As such, the partial information decomposition present in this paper is equivalent to the pointwise partial information decomposition presented in [27].

Let us now use the closed-form solution (75) from Theorem 5 to evaluate the partial information contents for the n=2 redundancy lattice of information contents. Starting from the bottom, we get the intersection information content,

(80) $$h_{\partial }\left(x⊓y\right)=h\left(x⊓y\right),$$

followed by the unique information contents,

(81) $$\begin{array}{cccc} h_{\partial }\left(x\right) & =h(x) & -h(x⊓y) & =h(x\backslash y), \end{array}$$

(82) $$\begin{array}{cccc} h_{\partial }\left(y\right) & =h(y) & -h(x⊓y) & =h(y\backslash x), \end{array}$$

and, finally, the synergistic information content,

(83) $$h_{\partial }\left(x,y\right)=h\left(x,y\right)-h\left(x⊔y\right)=h\left(x⊕y\right).$$

It is clear that these partial information contents recover the intersection, unique and synergistic information contents from Section 3 and Section 4. Moreover, by inserting these partial terms back into (73) for α={{x,y}}, we recover the earlier decomposition (35) of Johnny’s information,

(84) $$\begin{array}{c} h\left(x,y\right)=h_{\partial }\left(x⊓y\right)+h_{\partial }\left(x\right)+h_{\partial }\left(y\right)+h_{\partial }\left(x,y\right)=h\left(x⊓y\right)+h\left(x\backslash y\right)+h\left(y\backslash x\right)+h\left(x⊕y\right). \end{array}$$

Of course, our aim is to generalise this result such that we can decompose the joint information content for an arbitrary number of marginal realisations. This can be done by first evaluating the partial information contents over the redundancy lattice corresponding to *n* marginal realisations, and then subsequently inserting the results back into (73) for $\alpha =\{\left\{x_{1},x_{2},\ldots ,x_{n}\right\}\}$. For example, we can invert the n=3 redundancy lattice of information contents which yields the partial information contents shown in Figure 10. (The inversion is evaluated in the Appendix A.) When inserted back into (73), we get the following decomposition for Johnny’s information,

(85) $$\begin{array}{cc} h(x,y,z)\; & =h(x⊓y⊓z)\\ \; & \;+h\left((x⊓y)\backslash z\right)+h\left((x⊓z)\backslash y\right)+h\left((y⊓z)\backslash x\right)\\ \; & \;+h\left(x⊓(y⊕z)\right)+h\left(y⊓(x⊕z)\right)+h\left(z⊓(x⊕y)\right)\\ \; & \;+h\left(x\backslash (y,z)\right)+h\left(y\backslash (x,z)\right)+h\left(z\backslash (x,y)\right)+h\left((x⊕y)⊓(x⊕z)⊓(y⊕z)\right)\\ \; & \;+h\left((x⊕y)⊓(x⊕z)\backslash (y,z)\right)+h\left((x⊕y)⊓(y⊕z)\backslash (x,z)\right)+h\left((x⊕z)⊓(y⊕z)\backslash (x,y)\right)\\ \; & \;+h\left((x⊕y)\backslash ((x,z)⊔(y,z))\right)+h\left((x⊕z)\backslash ((x,y)⊔(y,z))\right)+h\left((y⊕z)\backslash ((x,y)⊔(x,z))\right)\\ \; & \;+h\left((x,y)⊕(x,z)⊕(y,z)\right). \end{array}$$

**Figure 11:**
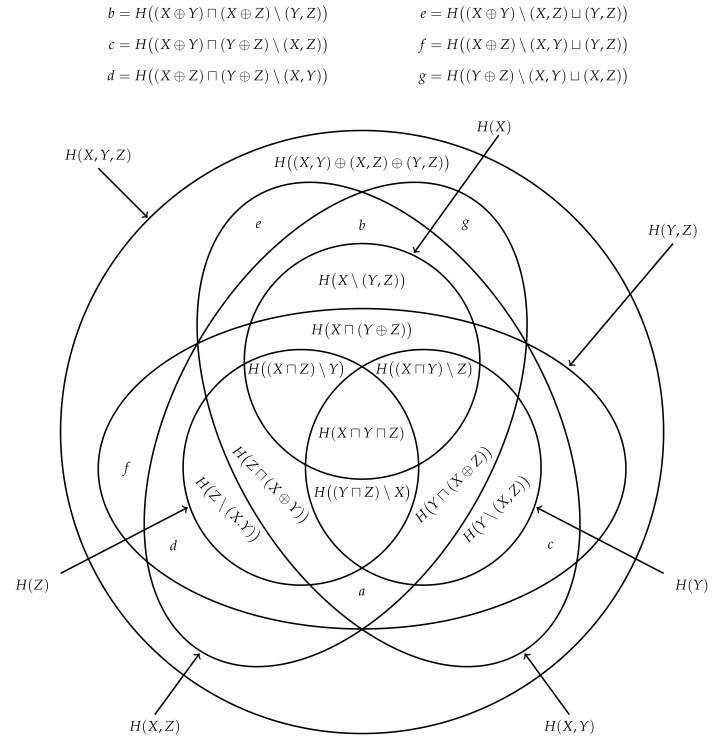
This Venn provides a visual representation of the decomposition of the joint entropy H(X,Y,Z). This decomposition is given by replacing *x*, *y*, *z* and *h* with *X*, *Y*, *Z* and *H* in (85), respectively.

Finally, we can also consider taking the expectation value of each term in the redundancy lattice of information contents. Since the expectation is a linear and monotonic operator, the resulting expectation values will inherit the structure of the redundancy lattice of information contents and so form a redundancy lattice of entropies, i.e., Figure 10 with *x*, *y*, *z* and *h* replaced by *X*, *Y*, *Z* and *H*, respectively. By inverting the n=2 redundancy lattice of entropies, we can recover the decomposition (40) from Figure 5. Furthermore, inverting the n=3 lattice generalises this result and is depicted in Figure 11.

## 8. Union and Intersection Mutual Information

Suppose that Alice, Bob and Johnny are now additionally and commonly observing the variable *Z*. When a realisation (x,y,z) occurs, Alice’s information for *z* is given by the conditional information content h(x|z), while Bob’s conditional information is given by h(y|z) and Johnny’s conditional information is given by h(x,y|z). By using the same argument as in Section 3, it is easy to see that Eve’s conditional information given *z* is given by the conditional union information content,

(86) $$h\left(x⊔y|z\right)=\max\left(h(x|z),h(y|z)\right).$$

Likewise, we can define the conditional unique information contents and conditional intersection information content, respectively,

(87) $$\begin{array}{cc} h(x\backslash y|z) & =h\left(x⊔y|z\right)-h\left(y|z\right)=\max\left(h(x|z)-h(y|z),0\right), \end{array}$$

(88) $$\begin{array}{cc} h(y\backslash x|z) & =h\left(x⊔y|z\right)-h\left(x|z\right)=\max\left(0,h(y|z)-h(x|z)\right), \end{array}$$

(89) $$\begin{array}{cc} h(x⊓y|z) & =h\left(x|z\right)+h\left(y|z\right)-h\left(x⊔y|z\right)=\min\left(h(x|z),h(y|z)\right). \end{array}$$

Furthermore, since Johnny’s conditional information h(x,y|z) is no less than Eve’s conditional information content h(x⊔y|z), we can also define the conditional synergistic information content,

(90) $$h\left(x⊕y|z\right)=h\left(x,y|z\right)-h\left(x⊔y|z\right)=\min\left(h(y|x,z),h(x|y,z)\right).$$

Similar to (35), we can decompose Johnny’s conditional information h(x,y|z) into the following components,

(91) h(x,y|z)=h(x⊔y|z)+h(x⊕y|z)=h(x⊓y|z)+h(x\y|z)+h(y\x|z)+h(x⊕y|z).

Moreover, similar to (38), the conditional mutual information content is equal to the difference between the conditional intersection information content and the conditional synergistic information content,

(92) i(x;y|z)=h(x|z)+h(y|z)−h(x,y|z)=h(x⊓y|z)−h(x⊕y|z).

Notice that all of the above definitions directly correspond to the definitions of the unconditioned quantities, with all probability distributions conditioned on *z* here.

Let us now consider how much information each of our observers have about the commonly observed realisation *z*. The information that Alice has about *z* from observing *x* is given by the mutual information content,

(93) i(x;z)=h(x)−h(x|z).

Similarly, Bob’s information about *z* is given by i(y;z), while Johnny’s information is given by the joint mutual information content i(x,y;z). Thus, the question naturally arises—are we able to quantify how much information Eve has about the realisation *z* from knowing Alice’s and Bob’s shared information?

Clearly, we could consider defining the union mutual information content,

(94) i(x⊔y;z)=h(x⊔y)−h(x⊔y|z).

It is important to note that, while the mutual information can be defined in three different ways i(x,z)=h(x)−h(x|z)=h(x)+h(z)−h(x,z)=h(z)−h(z|x), there is only one way in which one can define this function. (Indeed, this point aligns well with our argument based on exclusions presented in [28].) Similar to (94), we could consider respectively defining the unique mutual information contents, the intersection mutual information content and synergistic mutual information content,

(95) $$\begin{array}{cc} i(x\backslash y;z) & =h(x\backslash y)-h(x\backslash y|z), \end{array}$$

(96) $$\begin{array}{cc} i(y\backslash x;z) & =h(y\backslash x)-h(y\backslash x|z), \end{array}$$

(97) $$\begin{array}{cc} i(x⊓y;z) & =h(x⊓y)-h(x⊓y|z), \end{array}$$

(98) $$\begin{array}{cc} i(x⊕y;z) & =h(x⊕y)-h(x⊕y|z). \end{array}$$

As with the mutual information content (18), there is nothing to suggest that these quantities are non-negative. Of course, the mutual information or expected mutual information content (20) is non-negative. Thus, with this in mind, consider defining the union mutual information

(99) $$I\left(X⊔Y;Z\right)=E_{XYZ}\left[i(x⊔y;z)\right].$$

However, there is nothing to suggest that this function is non-negative. Consequently, it is dubious to claim that this function represents Eve’s expected information about *Z*, and is similarly fallacious to say that Eve’s information about *z* is given by the union mutual information content (94). Indeed, by inserting the definitions (21) and (86) into (94), it is easy to see why it is difficult to interpret these functions,

(100) $$\begin{array}{cc} i(x⊔y;z) & =\max\left(h(x),\;h(y)\right)-\max\left(h(x|z),\;h(y|z)\right)\\ \; & =\max\left(\min\left(i(x;z),\;h(x)-h(y|z)\right),\;\min\left(h(y)-h(x|z),\;i(y;z)\right)\right). \end{array}$$

That is, the union mutual information content can mix the information content provided by one realisation with the conditional information content provided by another. Thus, there is no guarantee that this function’s expected value will be non-negative. It is perhaps best to interpret this function as being a difference between two surprisals, rather than a function which represent information. Of course, similar to the multivariate mutual information (9), the union mutual information can be used a summary quantity provided one is careful not to misinterpret its meaning. The same is true for the unique mutual informations, intersection mutual information and synergistic mutual information, which we can similarly define,

(101) $$\begin{array}{cc} I(X\backslash Y;Z) & =E_{XYZ}\left[i(x\backslash y;z)\right], \end{array}$$

(102) $$\begin{array}{cc} I(Y\backslash X;Z) & =E_{XYZ}\left[i(y\backslash x;z)\right], \end{array}$$

(103) $$\begin{array}{cc} I(X⊓Y;Z) & =E_{XYZ}\left[i(x⊓y;z)\right], \end{array}$$

(104) $$\begin{array}{cc} I(X⊕Y;Z) & =E_{XYZ}\left[i(x⊕y;z)\right]. \end{array}$$

Despite lacking the clear interpretation that we had for the information contents, these functions share a similar algebraic structure. For example, by using (35) and (91), we can decompose the mutual information content into the following components,

(105) i(x,y;z)=i(x⊓y;z)+i(x\y;z)+i(y\x;z)+i(x⊕y;z),

which is similar to the earlier decomposition of the joint entropy (35). Moreover, similar to (38), by using (38) and (92), we get that the multivariate mutual information content is given by the difference between the intersection mutual information content and the synergistic mutual information content,

(106) $$\begin{array}{cc} i(x;y;z) & =i(x;y)-i(x;y|z)=h(x⊓y)-h(x⊕y)-h(x⊓y)+h(x⊕y|z)\\ \; & =i(x⊓y;z)-i(x⊕y;z). \end{array}$$

Of course, since the expectation value is a linear operator, both of these results can be carried over to the joint mutual information. Hence, the mutual information can be decomposed into the following components,

(107) I(X,Y;Z)=I(X⊓Y;Z)+I(X\Y;Z)+I(Y\X;Z)+I(X⊕Y;Z).

while the the multivariate mutual information is equal to the intersection mutual information minus the synergistic mutual information,

(108) $$\begin{array}{cc} I(X;Y;Z) & =I(X;Y)-I(X;Y|Z)=H(X⊓Y)-H(X⊕Y)-H(X⊓Y)+H(X⊕Y|Z)\\ \; & =I(X⊓Y;Z)-I(X⊕Y;Z). \end{array}$$

This latter result aligns with Williams and Beer’s prior result that the multivariate mutual information conflates redundant and synergistic information (Equation (14) [23]).

## 9. Conclusions

The main aim of this paper has been to understand and quantify the distinct ways that a set of marginal observers can share their information with some non-observing third party. To accomplish this objective, we examined the distinct ways in which two marginal observers, Alice and Bob, can share their information with the non-observing individual, Eve, and introduced several novel information-theoretic quantities: the union information content, which quantifies how much information Eve gets from the Alice and Bob; the intersection information content, which quantifies how much information Eve could have gotten from either Alice or Bob; and the unique information content, which quantifies how much information Eve gets from Alice relative to Bob, and vice versa. We then investigated the algebraic structure of these new measures of shared marginal information and showed that the structure of shared marginal information is that of a distributive lattice. Next, by using the fundamental theorem of distributive lattices, we showed that these new measures are isomorphic to the various unions and intersections of sets. This isomorphism is similar to Yeung’s correspondence between multivariate mutual information and signed measure [6,7]. However, in contrast to Yeung’s correspondence, the measures of information content presented in this paper are non-negative and maintain a clear operational meaning regardless of the number of realisations or variables involved. (This is, of course, excepting the mutual information contents presented in Section 8, which are not non-negative.)

The appearance of a lattice structure within the context of information theory is by no means novel. Han [12] developed a lattice-theoretic description of the entropy over a Boolean lattice generated by a set of random variables. This lattice encapsulates all linear sums and differences of the basic information-theoretic quantities, i.e., entropy, conditional entropy, mutual information and conditional mutual information. Moreover, this lattice structure captures several of the existing multivariate generalisations of mutual information [29], including the aforementioned multivariate mutual information (9) (which is also known as the interaction information [10], amount of information [2] or co-information [11]), the total correlation [30] (which is also known as the multivariate constraint [31], multi-information [32] or integration [33]), the dual total correlation [12] (which is also known as binding information [34]) and the novel measure of multivariate mutual information defined by Chan et al. [29] (see Han [12] and Chan et al. [29] for further details). Similar to the lattice of shared marginal information content, Han’s lattice is distributive—indeed, on a fundamental level, it is this algebraic structure that enables Yeung [6,7] to establish a correspondence with signed measure. Nevertheless, there two important differences to note between Han’s information lattice and the lattice of shared marginal information content: Firstly, Han’s lattice is based upon the entropies of random variables rather than the information content of realisations. In principle, there is no reason why one could not consider the information content of a Boolean lattice generated by a set of realisations (although the mutual information content would not be non-negative). Secondly, the Möbius inverse on Han’s information lattice yields the multivariate mutual information (9), which is not non-negative. In contrast, the partial information contents (75) that result from the Möbius inversion of the lattice of shared marginal information content are non-negative. Thus, in contrast to the multivariate mutual information, the new measures of multivariate information presented in this paper maintain their operational meaning for any number of random variables.

Similar to Han, Shannon [35] introduced his own information lattice, although it is based upon the notion of common information. In comparison to Shannon’s other work, this paper is not well recognised. Indeed, this common information was later independently proposed and studied by Gács and Körner [36]. Shannon’s original paper is relatively brief; however, Li and Chong [37] expanded upon Shannon’s discussion by formalising his argument in terms of σ-algebras and sample space partitions (see also [38]). To be specific, they described a random variable *X* as “being-richer-than” another random variable *Y* if the former’s sample space partition is finer than the latter’s sample space partition. Moreover, if their σ-algebras coincide, then two random variables are said to be informationally equivalent. This relation naturally forms a partial order over a set of random variables. For all *X* and *Y*, the joint variable (X,Y) is the poorest amongst all of the variables that are richer than both *X* and *Y*. Conversely, one can define a random variable *Z* that is the richest amongst all of the variables that are poorer than both *X* and *Y*. The entropy of this common variable *Z* defines the aforementioned common information. In contrast to the joint variable (X,Y), it is relatively difficult to characterise the common variable *Z* [36,37,39]. Nevertheless, its existence is sufficient for the definition of Shannon’s information lattice [35,37]. There are several features that distinguish this lattice from the lattice of shared marginal information. Firstly, similar to Han’s information lattice, the joint entropy and common information are defined in terms of entire random variables, rather than the information content of realisations. Secondly, even if we were to restrict ourselves to the comparing Shannon’s information lattice to the lattice of shared marginal entropy, the meet and join operations for these lattices are fundamentally different. We have already discussed the between their respective join operations, i.e., the joint entropy and union entropy, in Section 3 and Section 4. If we consider their respective meet operations, we get the common information is relatively restrictive compared to the intersection entropy, due to the fact that the common information requires one to identify the common random variable *Z*. This follows from the fact that the intersection information is greater than or equal the mutual information (43), which is in turn greater than or equal to the common information [36]. Finally, in general, Shannon’s information lattice is not distributive, nor is it even modular [35,37]. Thus, unlike the lattice of share marginal information or Han’s information lattice, the fundamental theorem of distributive lattice is not applicable, and hence Shannon’s information lattice does inherit any set-like identities.

The secondary objective of this paper has been to understand and demonstrate how we can use the measures of shared information content to decompose multivariate information. We began by comparing the union information content to the joint information content and used this comparison to define a measure of synergistic information content that captures how much more information a full joint observer, Johnny, has relative to an individual, Eve, who knows which joint realisation has occurred, but only knows the marginal distributions. We showed how one can use this measure, together with the measures of shared information content, to decompose the joint information content. We then compared the algebraic structure of joint information to the lattice structure of shared information, and showed how one can find the redundancy lattice from the partial information decomposition [23] embedded within this larger algebraic structure. More specifically, since this paper considers information contents, this redundancy lattice is actually same as the specificity lattice from pointwise partial information decomposition [27,28]. This observation connects the work presented in this paper to the existing body of theoretical literature on information decomposition [23,40,41,42,43,44,45,46,47,48,49,50,51,52,53,54,55,56,57,58,59,60,61,62], and its applications [63,64,65,66,67,68,69,70,71,72,73,74,75,76,77,78,79,80,81,82,83,84,85,86]. (For a brief summary of this literature, see [24].) Nevertheless, in contrast to the pointwise partial information decomposition [27,28], most of these approaches aim to decompose the average mutual information rather than the information content. The ability to decompose information content, and pointwise mutual information, provides a unique perspective on multivariate dependency.

To our knowledge, the only other approach that attempts to provide this pointwise perspective is due to Ince [87]. Ince’s approach proposes a method of information decomposition based upon the entropy, but can be applied to the information content (or in Ince’s terminology, the local entropy). Of particular relevance to this paper, Ince obtains a result that is equivalent to (38) whereby the mutual information content is equal to the redundant information content minus the synergistic information content (Equation (5) [87]). However, Ince’s definition of redundant information content differs from that of the intersection information content in (38). To be specific, it is based upon the sign of the multivariate mutual information content (or pointwise co-information), which is interpreted as a measure of “the set-theoretic overlap” of multiple information contents (or local entropies) (p. 7 [87]). However, as discussed in Section 1, this set-theoretic interpretation of the multivariate mutual information (co-information) is problematic. To account for these difficulties, Ince disregards the negative values, defining the redundant information content to equal to the multivariate mutual information when it is positive, and to be zero otherwise.

There are several avenues of inquiry for which this research will yield new insights, particularly in complex systems, neuroscience and communications theory. For instance, these measures might be used to better understand and quantify distributed intrinsic computation [66,79]. It is well known that that dynamics of individual regions in the brain depend synergistically on multiple other regions; synergistic information content might provide a means to quantify such dependencies in neural data [69,77,88,89,90]. Furthermore, these measures might be helpful for quantifying the synergistic encodings used in network coding [7]. Finally, it is well-known that many biological traits are not dependent on any one gene, but rather are synergistically dependent on two or more genes, and the decomposed information provides a means to quantify the unique, redundant and synergistic dependencies between a trait and a set of genes [91,92,93,94].

## Appendix A

We can use the closed-form (75) to evaluate the partial information contents for the n=3 redundancy lattice of information contents. Starting from the bottom and working up through the redundancy lattice, we have the following partial information contents:

(A1) $$h_{\partial }\left(x⊓y⊓z\right)\;=h(x⊓y⊓z);$$

(A2) $$\begin{array}{ll} h_{\partial }\left(x⊓y\right)\; & =h(x⊓y)-h(x⊓y⊓z)\\ & =h\left((x⊓y)\backslash z\right); \end{array}$$

(A3) $$\begin{array}{cc} h_{\partial }\left(x⊓(y,z)\right)\; & =h\left(x⊓(y,z)\right)-h\left((x⊓y)⊔(x⊓z)\right)\\ & =h\left(x⊓(y,z)\right)-h\left(x⊓(y⊔z)\right)\\ & =h\left(x⊓(y⊕z)\right); \end{array}$$

(A4) $$\begin{array}{cc} h_{\partial }\left(x\right)\; & =h\left(x\right)-h\left(x⊓(y,z)\right)\\ & =h\left(x\backslash (y,z)\right); \end{array}$$

(A8) $$\begin{array}{cc} h_{\partial }\left(x,y,z\right)\; & =h\left(x,y,z\right)-h\left((x,y)⊔(x,z)⊔(y,z)\right)\\ \; & =h\left((x,y)⊕(x,z)⊕(y,z)\right). \end{array}$$

**Lemma** **A1.**
*We have the following identity,*

(A9) $$h\left((x⊓y)\backslash z\right)=h\left((x\backslash z)⊓(y\backslash z)\right).$$

**Proof.** From (23) and (25), we have that

(A10) $$\begin{array}{cc} h\left((x⊓y)\backslash z\right)\; & =\max\left(\min(h(x),h(y))-h(z),0\right)=\max\left(\min(h(x)-h(z),\;h(y)-h(z)),0\right)\\ \; & =\min\left(\max(h(x)-h(z),0),\;\max(h(y)-h(z),0)\right)=h\left((x\backslash z)⊓(y\backslash z)\right), \end{array}$$

where we have used the fact that max(a,min(b,c)) is equal to min(max(a,b),max(a,c)).□

**Lemma** **A2.**
*We have the following identity,*

(A11) $$h\left(((x,y)\backslash (y,z))⊓((x,z)\backslash (y,z))\right)=h\left(x\backslash (y,z)\right)+h\left((x⊕y)⊓(x⊕z)\backslash (y,z)\right).$$

**Proof.** From (35), we have that

(A12) $$\begin{array}{cc} h\left((x,y)\backslash (y,z)\right)\; & =h\left((x⊓y)\backslash (y,z)\right)+h\left((x\backslash y)\backslash (y,z)\right)+h\left((y\backslash x)\backslash (y,z)\right)+h\left((x⊕y)\backslash (y,z)\right)\\ \; & =h\left(x\backslash (y,z)\right)+h\left((y\backslash x)\backslash (y,z)\right)+h\left((x⊕y)\backslash (y,z)\right), \end{array}$$

and since

(A13) $$h\left((y\backslash x)\backslash (y,z)\right)=\max\left(h(y\backslash x)-h(y,z),0\right)=0,$$

we get that

(A14) $$h\left((x,y)\backslash (y,z)\right)=h\left(x\backslash (y,z)\right)+h\left((x⊕y)\backslash (y,z)\right).$$

Finally, by inserting this result into (25), we get that

$$\begin{array}{ll} h(((x,y)\backslash (y,z)) & ⊓((x,z)\backslash (y,z))\\ & =\min\left(h\left(x\backslash (y,z)\right)+h\left((x⊕y)\backslash (y,z)\right),\;h\left(x\backslash (y,z)\right)+h\left((x⊕z)\backslash (y,z)\right)\right)\\ & =h\left(x\backslash (y,z)\right)+h\left(((x⊕y)\backslash (y,z))⊓((x⊕z)\backslash (y,z))\right).□ \end{array}$$

**Lemma** **A3.**
*We have the following identity,*

(A15) $$h\left(((x,y)\backslash ((x,z)⊔(y,z))\right)=h\left(((x⊕y)\backslash ((x,z)⊔(y,z))\right).$$

**Proof.** By (35), we have that

(A16) $$\begin{array}{cc} h\left((x,y)\backslash ((x.z)⊔(y,z))\right)\; & =h\left((x⊓y)\backslash ((x,z)⊔(y,z))\right)+h\left((x\backslash y)\backslash ((x,z)⊔(y,z))\right)\\ \; & \;+h\left((y\backslash x)\backslash ((x,z)⊔(y,z))\right)+h\left((x⊕y)\backslash ((x,z)⊔(y,z))\right). \end{array}$$

Since h(x⊓y), h(x\y) and h(y\x) are less than $h\left(((x,z)⊔(y,z)\right)$, from (23) we have that $h\left((x⊓y)\backslash ((x,z)⊔(y,z))\right)$, $h\left((x\backslash y)\backslash ((x,z)⊔(y,z))\right)$ and $h\left((y\backslash x)\backslash ((x,z)⊔(y,z))\right)$ are all equal to 0.□

**Lemma** **A4.**
*We have the following identity,*

(A17) $$h\left(x⊓(y\backslash x)\right)=0.$$

**Proof.** We have that

(A18) $$\begin{array}{c} h\left(x⊓(y\backslash x)\right)=h\left(x⊓(x,y)\right)-h\left(x⊓x\right)=h\left(x⊓x\right)-h\left(x\right)=0, \end{array}$$

where we have used (44) and (68).□

**Lemma** **A5.**
*We have the following identity,*

(A19) $$h\left((y\backslash x)⊓(y,z)\right)=h\left(y\backslash x\right).$$

**Proof.** We have that

(A20) $$\begin{array}{cc} h\left((y\backslash x)⊓(y,z)\right)\; & =h\left((y\backslash x)⊓(y\backslash x,y⊓x,z)\right). \end{array}$$

Using (68), this then reduces to

(A21) $$\begin{array}{c} h\left((y\backslash x)⊓(y,z)\right)=h\left(y\backslash x\right).□ \end{array}$$

**Lemma** **A6.**
*We have the following identity,*

(A22) $$h\left(x⊓(x⊕z)\right)=0.$$

**Proof.** We have

$$\begin{array}{cc} h\left(x⊓(x⊕z)\right)\; & =h\left(x⊓(x,z)\right)-h\left(x⊓x\right)+h\left(x⊓(z\backslash x)\right), \end{array}$$

which then reduces via (68), (44) and Lemma A4 to:

(A23) $$\begin{array}{cc} h\left(x⊓(x⊕z)\right)\; & =h\left(x\right)-h\left(x\right)+h\left(x⊓(z\backslash x)\right)+0\\ \; & =0.□ \end{array}$$

**Lemma** **A7.**
*We have the following identity,*

(A24) $$h\left((y\backslash x)⊓(x⊕z)\right)=h\left(y⊓(x⊕z)\right).$$

**Proof.** We have that

$$\begin{array}{cc} h\left(y⊓(x⊕z)\right)\; & =h\left((y\backslash x)⊓(x⊕z)\right)+h\left((y⊓x)⊓(x⊕z)\right). \end{array}$$

However, since $h\left(x⊓(x⊕z)\right)=0$ via Lemma A6 and therefore $h\left((y⊓x)⊓(x⊕z)\right)=0$, we have

$$\begin{array}{c} h\left(y⊓(x⊕z)\right)=h\left((y\backslash x)⊓(x⊕z)\right) \end{array}$$

as required.□

**Lemma** **A8.**
*We have the following identity,*

(A25) $$h\left((x⊕y)⊓(x,z)⊓(y,z)\right)=h\left(z⊓(x⊕y)\right)+h\left((x⊕y)⊓(x⊕z)⊓(y⊕z)\right).$$

**Proof.** We have that

(A26) $$\begin{array}{cc} h\left((x⊕y)⊓(x,z)⊓(y,z)\right)\; & =h\left((x⊕y)⊓x⊓(y,z)\right)+h\left((x⊕y)⊓(z\backslash x)⊓(y,z)\right)\\ \; & \;+h\left((x⊕y)⊓(x⊕z)⊓(y,z)\right). \end{array}$$

Then, by using Lemma A6, we get that

(A27) $$\begin{array}{cc} h\left((x⊕y)⊓(x,z)⊓(y,z)\right)\; & =h\left((x⊕y)⊓(z\backslash x)⊓(y,z)\right)+h\left((x⊕y)⊓(x⊕z)⊓(y,z)\right)\\ \; & =h\left((x⊕y)⊓(z\backslash x)⊓y\right)+h\left((x⊕y)⊓(z\backslash x)⊓(z\backslash y)\right)\\ \; & \;+h\left((x⊕y)⊓(z\backslash x)⊓(y⊕z)\right)+h\left((x⊕y)⊓(x⊕z)⊓y\right)\\ \; & \;+h\left((x⊕y)⊓(x⊕z)⊓(z\backslash y)\right)+h\left((x⊕y)⊓(x⊕z)⊓(y⊕z)\right)\\ \; & =h(\left(x⊕y\right)⊓\left(z\backslash x\right)⊓\left(z\backslash y\right)+h\left((x⊕y)⊓(x⊕z)⊓(y⊕z)\right), \end{array}$$

where we have used Lemma A6 four more times. Finally, using Lemma A7, we get that

(A28) $$\begin{array}{cc} h\left((x⊕y)⊓(x,z)⊓(y,z)\right)\; & =h\left((x⊕y)⊓z\right)+h\left((x⊕y)⊓(x⊕z)⊓(y⊕z)\right), \end{array}$$

as required.□

**Lemma** **A9.**
*We have the following identity,*

(A29) $$\begin{array}{cc} h\left((x,y)⊓(x,z)⊓(y,z)\right)\; & =h\left(x⊓(y,z)\right)+h\left(y⊓(x⊕z)\right)+h\left(z⊓(x⊕y)\right)+h\left((y⊓z)\backslash x\right)\\ \; & \;+h\left((x⊕y)⊓(x⊕z)⊓(y⊕z)\right). \end{array}$$

**Proof.** We have that

(A30) $$\begin{array}{cc} h\left((x,y)⊓(x,z)⊓(y,z)\right)\; & =h\left(x⊓(x,z)⊓(y,z)\right)+h\left((y\backslash x)⊓(x,z)⊓(y,z)\right)+h\left((x⊕y)⊓(x,z)⊓(y,z)\right)\\ \; & =h\left(x⊓(y,z)\right)+h\left((y\backslash x)⊓(x,z)\right)+h\left((x⊕y)⊓(x,z)⊓(y,z)\right), \end{array}$$

where we have used (68) and Lemma A5. Next, we have that

(A31) $$\begin{array}{cc} h\left((x,y)⊓(x,z)⊓(y,z)\right)\; & =h\left(x⊓(y,z)\right)+h\left((y\backslash x)⊓x\right)+h\left((y\backslash x)⊓(z\backslash x)\right)+h\left((y\backslash x)⊓(x⊕z)\right)\\ \; & \;+h\left((x⊕y)⊓(x,z)⊓(y,z)\right)\\ \; & =h\left(x⊓(y,z)\right)+0+h\left((y⊓z)\backslash x\right)+h\left(y⊓(x⊕z)\right)\\ \; & \;+h\left((x⊕y)⊓(x,z)⊓(y,z)\right) \end{array}$$

where we have used Lemma A1, Lemma A4 and Lemma A7. Finally, we have that

(A32) $$\begin{array}{cc} h\left((x,y)⊓(x,z)⊓(y,z)\right)\; & =h\left(x⊓(y,z)\right)+h\left((y⊓z)\backslash x\right)+h\left(y⊓(x⊕z)\right)+h\left(z⊓(x⊕y)\right)\\ \; & \;+h\left((x⊕y)⊓(x⊕z)⊓(y⊕z)\right), \end{array}$$

where we have used via Lemma A8.□
